# Regulation of RIPK1 activation by TAK1-mediated phosphorylation dictates apoptosis and necroptosis

**DOI:** 10.1038/s41467-017-00406-w

**Published:** 2017-08-25

**Authors:** Jiefei Geng, Yasushi Ito, Linyu Shi, Palak Amin, Jiachen Chu, Amanda Tomie Ouchida, Adnan Kasim Mookhtiar, Heng Zhao, Daichao Xu, Bing Shan, Ayaz Najafov, Guangping Gao, Shizuo Akira, Junying Yuan

**Affiliations:** 1 Department of Cell Biology, Harvard Medical School, 240 Longwood Ave, Boston, MA 02115 USA; 2 Interdisciplinary Research Center on Biology and Chemistry, Shanghai Institute of Organic Chemistry, Chinese Academy of Sciences, 26 Qiuyue Rd, PuDong District, Shanghai, 201210 China; 3 Horae Gene Therapy Center and Vector Core, and Department of Physiological Systems, University of Massachusetts Medical School, 368 Plantation Street, AS6-2049, Worcester, MA 01605 USA; 4 Laboratory of Host Defense, WPI Immunology Frontier Research Center (IFReC), Osaka University, 3-1 Yamadaoka, Suita, Osaka 565-0871 Japan

## Abstract

Stimulation of TNFR1 by TNFα can promote three distinct alternative mechanisms of cell death: necroptosis, RIPK1-independent and -dependent apoptosis. How cells decide which way to die is unclear. Here, we report that TNFα-induced phosphorylation of RIPK1 in the intermediate domain by TAK1 plays a key role in regulating this critical decision. Using phospho-Ser321 as a marker, we show that the transient phosphorylation of RIPK1 intermediate domain induced by TNFα leads to RIPK1-independent apoptosis when NF-κB activation is inhibited by cycloheximide. On the other hand, blocking Ser321 phosphorylation promotes RIPK1 activation and its interaction with FADD to mediate RIPK1-dependent apoptosis (RDA). Finally, sustained phosphorylation of RIPK1 intermediate domain at multiple sites by TAK1 promotes its interaction with RIPK3 and necroptosis. Thus, absent, transient and sustained levels of TAK1-mediated RIPK1 phosphorylation may represent distinct states in TNF-RSC to dictate the activation of three alternative cell death mechanisms, RDA, RIPK1-independent apoptosis and necroptosis.

## Abstract (web summary)

TNFα can promote three distinct mechanisms of cell death: necroptosis, RIPK1-independent and dependent apoptosis. Here the authors show that TNFα-induced phosphorylation of RIPK1 in the intermediate domain by TAK1 plays a key role in regulating this decision.

## Introduction

RIPK1, a member of the receptor-interacting protein (RIP) serine-threonine kinase family, has emerged as a key upstream regulator that controls multiple downstream signaling pathways of TNFR1^1, 2^. Within minutes after cells stimulated by TNFα, RIPK1 is recruited into the TNFR1 signaling complex (TNF-RSC, also called complex I) together with signaling molecules such as TRADD, TRAF2 and cIAP1/2 to decide if a cell and ultimately, an organism, may live or die through apoptosis or necroptosis. Apoptosis may be mediated by binding of RIPK1, independent of its kinase activity, with FADD, an adaptor protein for caspase-8, which in turn promotes the activation of caspase-8 and executes apoptosis by triggering mitochondrial damage and the cleavage of downstream caspases such as caspase-3. Under apoptotic deficient conditions, RIPK1 may be activated to promote necroptosis by interacting with RIPK3 which in turn promotes the phosphorylation of MLKL to mediate the execution of necroptosis.

Ubiquitination of RIPK1 by cIAP1/2 in TNF-RSC is involved in mediating the activation of NF-κB by recruiting TAB1/2 to promote the activation of the TAK1 (transforming growth factor-β-activated kinase 1, also called MAP3K7)^3^. Activated TAK1 mediates the phosphorylation of IKKβ to promote the formation of the IKK complex consisting of IKKα/β/γ(NEMO)^4^. Although the best characterized function of TAK1 and the IKK complex including NEMO is to mediate the activation of NF-κB pathway, recent studies have unveiled that deficiencies in TAK1, NEMO, IKKα/β or the loss of cIAP1/2 can sensitize cells to RIPK1-dependent apoptosis (RDA) independently of their roles in NF-κB activation^5, 6^. On the other hand, in cells deficient for A20, an important ubiquitin-editing enzyme for RIPK1, or TAB2, which regulates the activation of TAK1, RIPK1 may be activated to interact with RIPK3 to mediate necroptosis^7, 8^. It is not clear, however, how activated RIPK1 might be directed to mediate two alternative modes of cell death, RDA or necroptosis, that both occur in a RIPK1 kinase-dependent manner.

RIPK1 contains an N-terminal kinase domain, an intermediate domain and a C-terminal death domain^1^. The kinase activity of RIPK1 may be activated upon stimulation of TNFR1 by TNFα under selective conditions, which leads to multiple deleterious consequences including cell death and inflammation. Inhibition of RIPK1 kinase activity using improved necrostatin-1 (R-7-Cl-O-Nec-1, Nec-1s), a highly specific small molecule inhibitor of RIPK1, and the use of RIPK1 kinase-dead mutant mice, have shown efficacy in a wide range of animal models of human diseases^9–11^. Small molecule inhibitors of RIPK1 are under clinical and preclinical development targeting human diseases. However, the molecular mechanism that controls the activation of RIPK1 kinase activity remains unclear.

Here we show that the intermediate domain of RIPK1 is phosphorylated transiently by TAK1 upon TNFα stimulation in wild-type (WT) cells in vitro and in vivo. While Ser321 (S321) phosphorylation of RIPK1 by TAK1 has no effect on the NF-κB activation, the loss of S321 phosphorylation promotes the binding of RIPK1 to FADD and RDA. On the other hand, the sustained TAK1-mediated phosphorylation of RIPK1 in multiple sites of the intermediate domain including S321 promotes its interaction with RIPK3 to mediate necroptosis. Our results elucidate the molecular mechanism of interaction between TAK1 and RIPK1, two critical mediators in the TNFα signaling pathway, distinct from their roles in the activation of the NF-κB pathway, and the mechanism by which the levels of RIPK1 phosphorylation control the cellular choices for alternative cell death mechanisms.

## Results

### Transient RIPK1 S321 phosphorylation upon TNFα stimulation

**Fig. 1:**
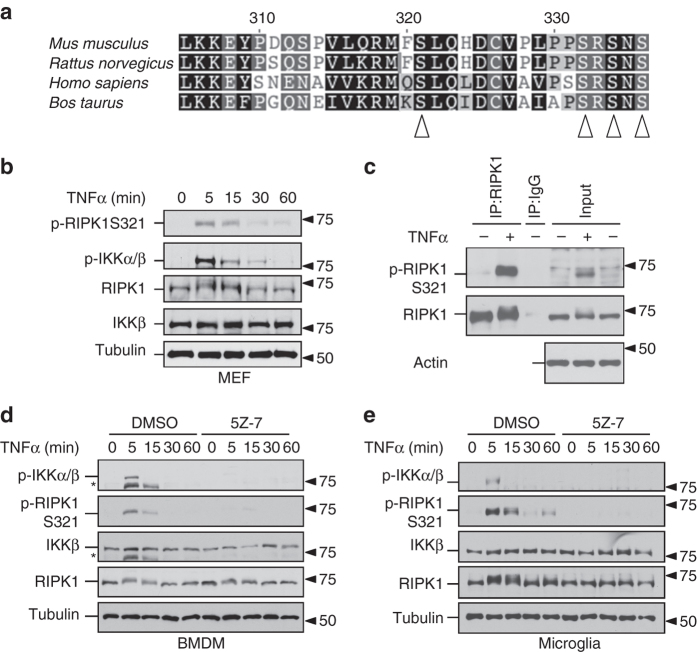
TNFα induces RIPK1 phosphorylation at S321. **a** Alignment of amino acid sequences in the relevant part of RIPK1 intermediate domain from indicated mammalian species. S321, S332, S334 and S336 as marked by arrowheads are highly evolutionarily conserved. **b** S321 of RIPK1 is transiently phosphorylated after TNFα treatment. MEF cells were treated with TNFα (10 ng/ml) and the samples were collected at indicted time-points. The cell lysates were analyzed by western blot. **c** Anti-p-S321-RIPK1 antibody specifically recognizes RIPK1 S321 phosphorylation. L929 cells were treated with TNFα (20 ng/ml) for 5 min. The cell lysates were subjected to immunoprecipitation by either RIPK1 antibody or IgG. Both lysates and IP samples were examined by western blot using anti-p-S321-RIPK1 and RIPK1 total antibodies. **d**, **e** TNFα induces RIPK1 S321 phosphorylation in murine primary BMDM and microglia cells. BMDM **d** and primary microglia **e** were pre-incubated with DMSO or TAK1 inhibitor, 5Z-7 (0.5 µM) for 30 min and treated with TNFα (10 ng/ml). Samples were collected at indicated time-points. *, nonspecific bands

S321 of RIPK1 was found to be phosphorylated in the kidney, lung and spleen tissues of mice under normal conditions in a global phosphoproteomic study and when expressed in 293T cells^11, 12^. S321 site is evolutionarily conserved in RIPK1 proteins from species including mouse, human, rat and cattle (Fig. 1a). S321 is located in a conserved sequence RMFSLQHDCV in murine RIPK1, or RMQSLQLDCV in human RIPK1. The +1 residue of this peptide is a ‘Leu’, which is also found in +1 residue of S177 in IKKβ known to be phosphorylated by TAK1^13^.

To confirm and characterize the significance of S321 phosphorylation, we developed a phospho-specific antibody against p-S321 of mouse RIPK1 (anti-p-S321-RIPK1). We probed western blots of cell lysates from mouse embryonic fibroblast (MEF) cells stimulated with TNFα for different periods of time. After stimulation of TNFα, the RIPK1 band showed a specific up-shift within 5 min TNFα stimulation and the shift reduced after 30–60 min treatment. This shifted band was recognized by anti-p-S321-RIPK1 (Fig. 1b). The phosphorylation of IKKβ, known to be mediated by TAK1^13^, was also detected during the same time frame. Induction of RIPK1 S321 phosphorylation was also found in RGC-5 cells stimulated by TNFα (Supplementary Fig. 1a). To confirm that the band recognized by anti-p-S321-RIPK1 is RIPK1, we immunoprecipitated RIPK1 from cells stimulated by TNFα and probed the immunocomplexes with anti-p-S321-RIPK1. As shown in Fig. 1c, anti-p-S321-RIPK1 specifically recognized immunoprecipitated RIPK1 from TNFα-stimulated cells but not from control cells. In addition to MEF and RGC-5 cells, we verified TNFα induced RIPK1 phosphorylation in primary cells. As that observed in MEFs and RGC-5 cells, in bone marrow-derived macrophages (BMDM) and mouse primary microglia stimulated by TNFα, the phosphorylation of RIPK1 S321 occurred in a similar pattern as that of IKKβ (Fig. 1d, e). Thus, S321 of RIPK1 is phosphorylated transiently after TNFα stimulation.

### TAK1 phosphorylates RIPK1 S321

**Fig. 2:**
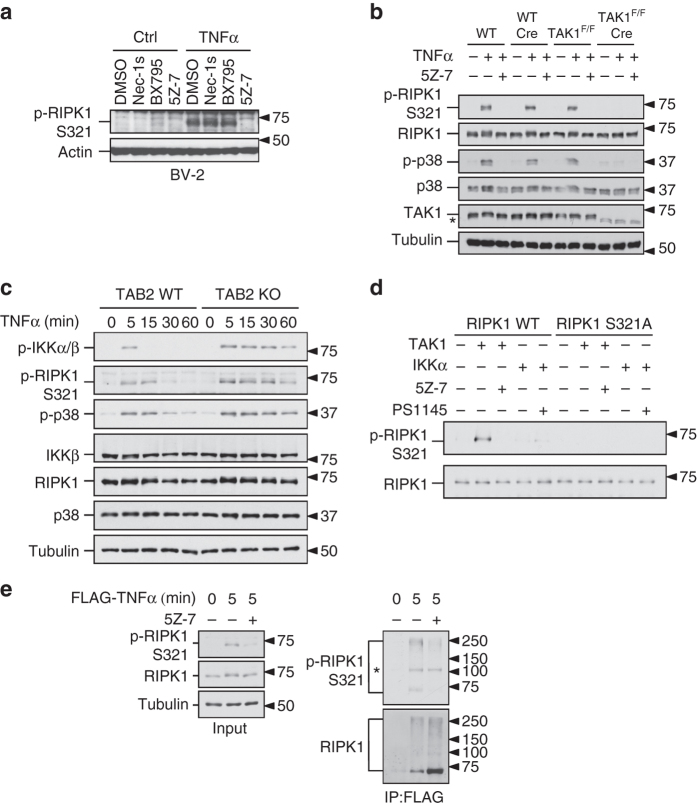
TAK1 phosphorylates RIPK1 at S321. **a** TNFα-induced RIPK1 S321 phosphorylation is inhibited by TAK1 inhibitor. BV-2 cells were treated by TNFα (20 ng/ml) together with DMSO, Nec-1s (10 µM), TBK1 inhibitor, BX795 (1 µM) and 5Z-7 (0.5 µM). **b** TAK1 is required for RIPK1 S321 phosphorylation. WT and TAK1^F/F^ MEFs with or without Cre expression were treated with TNFα (10 ng/ml) and 5Z-7 (0.5 µM) for 15 min and examined by indicated antibodies. **c** Hyperphosphorylation of RIPK1 S321 in TAB2 KO MEF. TAB2 WT and KO MEFs were treated with TNFα (10 ng/ml) and the samples were collected at indicated time-points. **d** FLAG-RIPK1 WT and S321A were expressed and purified from 293T cells and incubated with recombinant TAK1-TAB1 or GST-IKKα or their inhibitors, 5Z-7 or PS1145, respectively. The products were analyzed by western blot using indicated antibodies. **e** RIPK1 S321 phosphorylation in TNF-RSC. MEF cells were treated with FLAG-TNFα (50 ng/ml) for 5 min and TNF-RSC was purified by anti-FLAG immunoprecipitation. TNF-RSC immunocomplexes were analyzed by western blot using indicated antibodies. *, nonspecific bands

On the basis of the temporal profile of RIPK1 S321 phosphorylation upon stimulation by TNFα, we focused on characterizing the involvement of TAK1 and TBK1 as possible kinases responsible for phosphorylating RIPK1 S321 in response to TNFα stimulation. We treated L929 and BV-2 cells with TNFα in the presence of TAK1 inhibitor (5Z-7-Oxozeaenol, 5Z-7) or TBK1 inhibitor (BX795). The treatment with either TAK1 or TBK1 inhibitors blocked TNFα-stimulated S321 phosphorylation in L929 cells (Supplementary Fig. 1b), but only TAK1 inhibitor blocked the phosphorylation of S321 in TNFα-stimulated BV-2 cells (Fig. 2a). 5Z-7 also inhibited phosphorylation of RIPK1 S321 in RGC-5, MEF, primary BMDM and microglia cells stimulated by TNFα (Supplementary Fig. 1a, c and Fig. 1d, e). To determine whether TBK1 might mediate the phosphorylation of RIPK1 S321, we used CRISPR-Cas9 technology to generate TBK1 knockout (KO) BV-2 and MEF cells and then stimulated the cells with TNFα. We found that the phosphorylation of RIPK1 S321 was not blocked, but rather enhanced by TBK1 deficiency in both MEF and BV-2 cells (Supplementary Fig. 1c, d). Therefore, we conclude that TBK1 is not the kinase responsible for TNFα-induced RIPK1 S321 phosphorylation.

To provide definitive evidence for the role of TAK1 in mediating phosphorylation S321 of RIPK1, we obtained MEFs from mice homozygous for TAK1^flox/flox^ allele (TAK1^F/F^)^14^. To generate TAK1-deficient MEFs, we excised the floxed genomic fragment by infecting TAK1^F/F^ MEFs with virus expressing Cre and selected single colonies lacking the expression of TAK1 (TAK1^F/F^ Cre). In TAK1^F/F^ Cre MEFs, RIPK1 S321 phosphorylation in response to TNFα was substantially abolished (Fig. 2b). In contrast, the expression of Cre in WT MEF cells did not affect the expression level of TAK1 or RIPK1 S321 phosphorylation in response to TNFα (Fig. 2b), suggesting the blocked RIPK1 S321 phosphorylation in TAK1^F/F^ Cre MEFs was not a result of virus infection or Cre expression per se. Consistently, the phosphorylation levels of p38 MAPK, a downstream target of TAK1^15^, were also reduced in TAK1^F/F^ Cre MEFs but no in WT Cre MEFs (Fig. 2b).

TAK1 activation in response to TNFα is transient, which peaks at 5–10 min and decreases by TAB2-dependent recruitment of protein phosphatases^16^. Deficiency in TAB2 leads to sustained TAK1 activation and sensitized cells to necroptosis^8, 16^. We therefore characterized RIPK1 S321 phosphorylation in WT and TAB2 KO MEFs. We found that compared to that of WT MEFs, the phosphorylation of RIPK1 S321 was enhanced and sustained longer at later time-points in TAB2 KO MEFs when the signals in WT MEFs were already subsided (Fig. 2c). Consistent with the prolonged activation of TAK1, TNFα stimulation in TAB2 KO MEFs also led to sustained phosphorylation of p38 and IKKβ than that in WT cells.

Next we examined the ability of TAK1 to directly phosphorylate RIPK1 by in vitro kinase assay. To minimize the effect of auto-phosphorylation, we isolated kinase-dead RIPK1 protein, RIPK1 K45M, as a substrate and incubated it with recombinant TAK1 in the presence of ^32^P-ATP. When RIPK1 K45M was incubated alone, only minimal level of phosphorylation could be detected. When incubated with recombinant TAK1, RIPK1 was phosphorylated and the phosphorylation was blocked in the presence of TAK1 inhibitor (Supplementary Fig. 1e). Kinase-dead IKKβ, a well characterized substrate of TAK1 was used as a positive control. Phosphorylation of kinase-dead RIPK1 protein with additional S321A mutation by TAK1 in radioactive kinase assay was reduced but not eliminated (Supplementary Fig. 1e), suggesting the additional TAK1 phosphorylation sites on RIPK1. To further confirm that the TAK1-mediated phosphorylation occurred on S321, we purified RIPK1 WT and S321A mutant proteins and examined whether they could be phosphorylated by TAK1 in in vitro kinase assay. After incubation with recombinant TAK1, only RIPK1 WT, but not RIPK1 S321A mutant, could be recognized by anti-p-S321-RIPK1 antibody and the phosphorylation was inhibited in the presence of 5Z-7 (Fig. 2d). Thus, TAK1 can directly phosphorylate RIPK1 S321 in vitro.

Recently it was reported that IKKα/β could phosphorylate RIPK1^5^. Inhibition of TAK1 by 5Z-7 also blocked the activation of IKKβ occurring downstream of TAK1 in response to TNFα stimulation as expected (Supplementary Fig. 2a). To test the possible contribution of IKKα/β to RIPK1 S321 phosphorylation, we examined the effect of IKKα/β inhibitor, BMS345541, on RIPK1 S321 phosphorylation. The presence of BMS345541 suppressed IκBα phosphorylation, which is known to be mediated by IKKα/β kinase (Supplementary Fig. 2b)^17^. On the other hand, the levels of phospho-RIPK1 S321 and phospho-IKKβ were sustained even longer in presence of BMS345541 and TNFα (Supplementary Fig. 2b). In addition, we confirmed that recombinant IKKα could phosphorylate RIPK1 in in vitro kinase assay with ^32^P-ATP (Supplementary Fig. 2c) but the phosphorylated RIPK1 could not be recognized by anti-p-S321-RIPK1 antibody (Fig. 2d). Taken together, these results suggest that IKK complex is not involved in mediating the phosphorylation of RIPK1 S321.

The time-course study showed that phosphorylation of RIPK1 S321 occurred within 5 min of TNFα treatment, similar to that of TAK1-mediated IKKβ phosphorylation (Fig. 1b, d, e). Since TAK1 is transiently recruited to TNF-RSC within 5 min after TNFα treatment, we next tested if the phosphorylation of RIPK1 S321 occurred in TNF-RSC. MEF cells were treated with FLAG-tagged TNFα and TNF-RSC was purified by anti-FLAG immunoprecipitation. Consistent with the phosphorylation by TAK1, we detected the S321 phosphorylation of both high molecular weight ubiquitinated and un-ubiquitinated RIPK1 species in TNF-RSC, which was inhibited by 5Z-7 (Fig. 2e). Taken together, we conclude that TAK1 mediates the phosphorylation of RIPK1 S321 in TNF-RSC upon TNFα stimulation.

### S321 phosphorylation of RIPK1 requires cIAP1/2

**Fig. 3:**
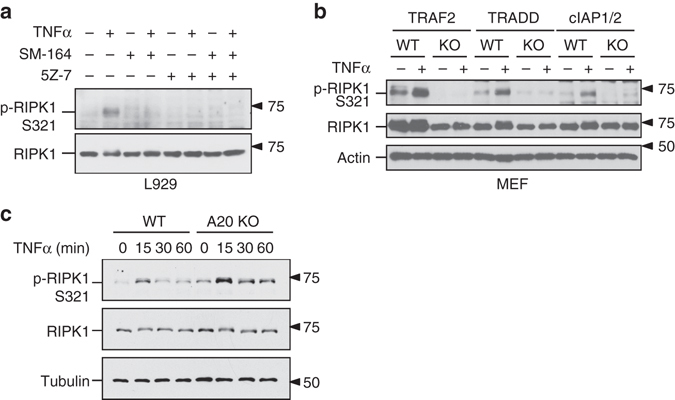
Ubiquitination of RIPK1 is essential for its phosphorylation by TAK1. **a** SM-164 treatment blocks RIPK1 S321 phosphorylation in L929 cells. The cells were pre-treated by SM-164 (50 nM) and/or 5Z-7 (0.5 µM) as indicated for 30 min, and incubated with TNFα (10 ng/ml) for 5 min. **b** RIPK1 S321 is not phosphorylated in the absence of TRAF2, TRADD or cIAP1/2. TRAF2, TRADD, cIAP1/2 KO MEFs and their corresponding WT MEFs were treated with TNFα for 5 min. **c** RIPK1 S321 hyperphosphorylation in A20 KO MEFs. A20 KO and WT MEFs were treated with TNFα and samples were collected at indicated time-points. The phosphorylation of RIPK1 S321 was analyzed by western blot using indicated antibodies **a**–**c**

cIAP1-mediated K63 ubiquitination of RIPK1 is known to be critical for the recruitment of TAK1 into the TNF-RSC upon TNFα stimulation^18^. To examine the role of K63 ubiquitination on RIPK1 in TNFα-stimulated S321 phosphorylation, we treated cells with SM-164, a small molecule IAP antagonist that can promote the degradation of cIAP1/2^19^. TNFα-induced RIPK1 ubiquitination in TNF-RSC was substantially reduced in the presence of SM-164 (Supplementary Fig. 3a). Accordingly, TNFα-induced RIPK1 S321 phosphorylation was abolished after SM-164 treatment (Fig. 3a). Furthermore, S321 phosphorylation was not detected in TNFα-stimulated cIAP1/2 double knockout (DKO) cells (Fig. 3b). Thus, cIAP1/2 is required for phosphorylation of RIPK1 S321 induced by TNFα.

To further characterize the requirement for RIPK1 ubiquitination in TAK1-mediated phosphorylation of S321, we examined the requirement of TRADD, which is involved in the recruitment of cIAP1/2 and TRAF2 to TNFR1, and TRAF2, an adaptor protein for the recruitment of cIAP1/2^20^. As shown in Fig. 3b, the phosphorylation of S321 stimulated by TNFα was not detectable in TRADD KO, or TRAF2 KO MEF cells. Consistent with the requirement of K63 ubiquitination in phosphorylation of S321, treatment with TAK1 inhibitor in the presence of SM-164 did not further sensitize cells to TNFα-induced cell death (Supplementary Fig. 3b). Taken together, these results suggest that cIAP1-mediated K63 ubiquitination of RIPK1 is important for promoting phosphorylation of RIPK1 S321.

Since A20 is a critical ubiquitin-editing enzyme that can move K63 ubiquitin chain from RIPK1 in TNFα-stimulated cells to down-regulate TNFα signaling^21^, we examined the role of A20 in regulating S321 phosphorylation of RIPK1. We found that TNFα-induced RIPK1 S321 phosphorylation in A20-deficient cells was significantly higher and persisted longer than that in WT cells (Fig. 3c). Given the fact that A20-deficient cells are hypersensitive to RIPK1 activation and necroptosis^22^, this result suggests that the possibility of elevated phosphorylation on RIPK1 by TAK1 may regulate the activation of RIPK1.

### Phosphorylation of RIPK1 S321 regulates RDA

**Fig. 4:**
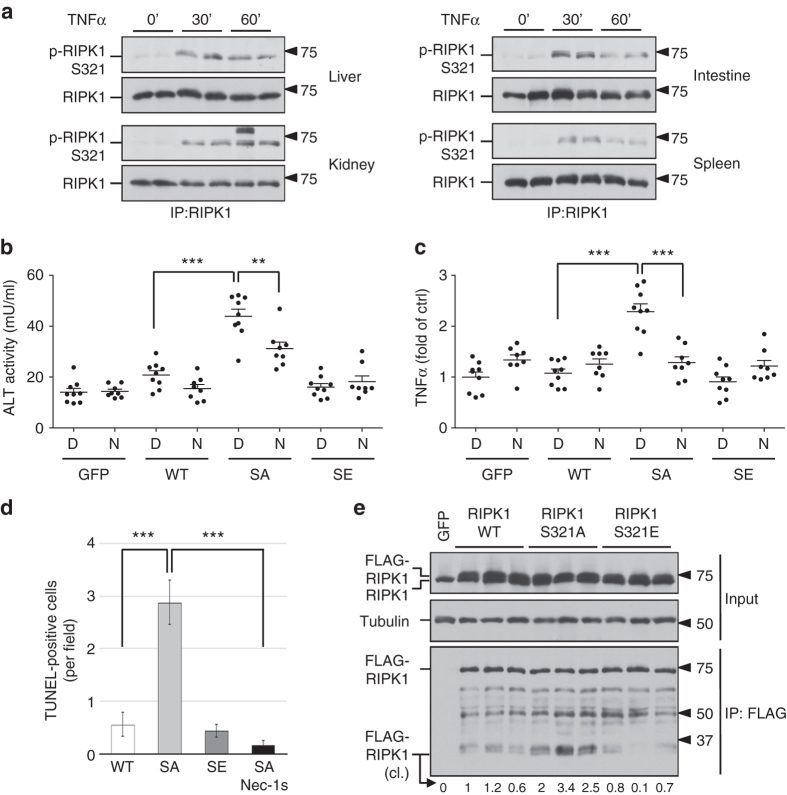
AAV-mediated RIPK1 S321A mutant expression induces liver damage in vivo. **a** Phosphorylation of RIPK1 S321 in mice tissues after TNFα injection. 30 µg TNFα were delivered to mice (8-week-old male) through intraperitoneal injections. Tissues were collected at 30 and 60 min after the injection. RIPK1 was immunoprecipitated from tissue lysates by RIPK1 total antibody. **b**, **c** Liver damage in mice induced by AAV-mediated RIPK1 S321A mutant expression. AAVs carrying RIPK1 WT, S321A, S321E or GFP as a control were delivered through tail vein injection. Sixteen days after the AAV injection, the mice were treated with DMSO (D, *n* = 9) or Nec-1s (N, *n* = 8) through oral dosing. Four weeks after the AAV injection, plasma and liver samples were collected. ALT **b** and TNFα ELISA **c** assays were performed as manufacture’s instructions. **d** TUNEL staining for assessing liver damage induced by AAV-mediated RIPK1 expression. Liver sections were stained with TUNEL and Hoechst. Cells with co-localized TUNEL and Hoechst signals were counted as TUNEL-positive. **e** AAV-mediated expression of FLAG-RIPK1 variants in liver. The liver lysates were subjected to anti-FLAG immunoprecipitation to differentiate endogenous RIPK1 and ectopic FLAG-RIPK1. Relative intensity of cleaved (cl.) RIPK1 bands was quantified. Error bar, s.e.m. **, *t*-test *P* < 0.01, ***P < 0.001. Three independent repeats were included in each data point

Next, we explored the biological significance of RIPK1 S321 phosphorylation. To determine whether RIPK1 S321 phosphorylation occurs in vivo, we stimulated mice with TNFα via intraperitoneal injections and characterized the phosphorylation of RIPK1 by western blot. We found that TNFα stimulation in vivo was able to induce the phosphorylation of RIPK1 S321 in the liver, kidney, intestine and spleen (Fig. 4a). To determine the significance of RIPK1 S321 phosphorylation in vivo, we compared the effects of virally transduced RIPK1 WT, S321A (SA) and S321E (SE) expression in the liver, delivered using adeno-associated virus (AAV) vector, a small single-stranded DNA-containing nonpathogenic human parvovirus that has been used as an efficient vehicle for gene transfer to different tissues including liver without apparent vector-related toxicities^23^. Specifically, we constructed AAV vectors expressing FLAG-tagged RIPK1 WT, S321A and S321E under the control of a liver-specific promoter TBG^24^. We followed the effect of RIPK1 expression in C57BL/6 mice intravenously injected with RIPK1 expression AAVs using plasma levels of alanine aminotransferase (ALT), a well-established biomarker for liver damage. C57BL/6 mice are known to be tolerogenic to rAAV gene delivery to the liver^23^. Only a basal level of ALT release was found in the plasma of mice received control (GFP-expressing) AAV. Interestingly, while low levels of ALT release were detected in the plasma of mice transduced with RIPK1 WT, significantly elevated levels of ALT were found in the plasma of mice that received RIPK1 S321A, but not S321E, AAV (Fig. 4b). Furthermore, the levels of TNFα were also significantly higher in the liver tissues of mice that received RIPK1 S321A virus (Fig. 4c). Importantly, the increased release of ALT and TNFα was inhibited in mice treated with Nec-1s (Fig. 4b, c). The effect of RIPK1 S321A virus to induce liver damage was directly verified using TUNEL staining (Fig. 4d). The expression level of FLAG-RIPK1 S321A was comparable to those of WT and S321E, suggesting their different physiological effect was not due to variations in expression levels (Fig. 4e). The cleavage of RIPK1 S321A was elevated than that of WT in vivo, while the cleavage of RIPK1 S321E was reduced (Fig. 4e). Thus, the expression of S321A was more effective than that of RIPK1 WT or S321E in inducing RIPK1-dependent liver damage and inflammation in vivo.

**Fig. 5:**
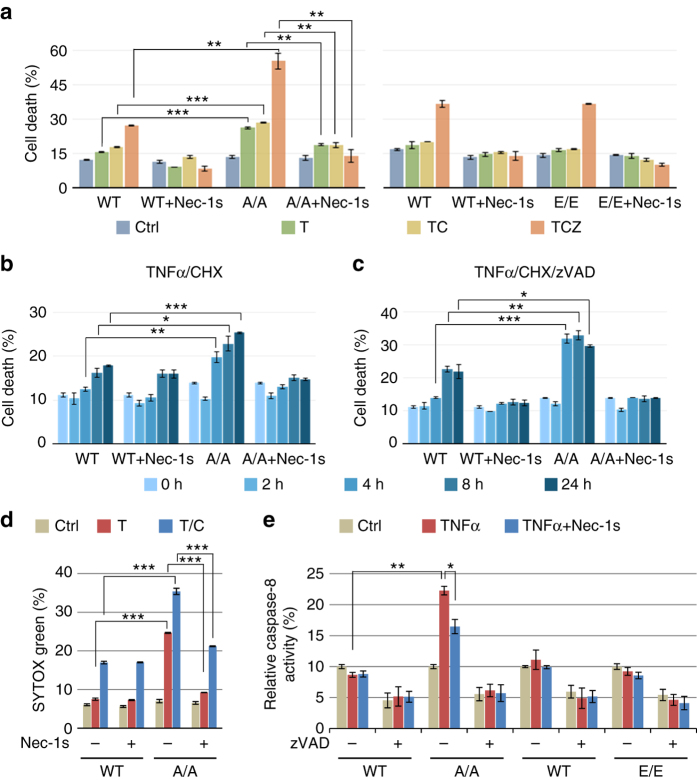
RIPK1 S321A mutation sensitizes cells to RIPK1-dependent cell death. **a**–**c** RIPK1 S321A(A/A) but not S321E(E/E) MEFs are more sensitive to RIPK1-dependent cell death. Immortalized MEFs generated from littermates of WT and RIPK1 S321A(A/A), or WT and S321E (E/E) were treated with TNFα (10 ng/ml), CHX (0.5 µg/ml), zVAD (20 µM) and Nec-1s (10 µM) as indicated. Cell death was measured by ToxiLight assay after 16 h treatment **a** or at indicated time-points **b**, **c** and normalized to TX-100-treated cells. **d** TNFα alone or TNFα/CHX induces RDA in RIPK1 S321A(A/A) mutant MEFs. WT and RIPK1 S321A(A/A) MEFs were treated with TNFα (10 ng/ml) or CHX (0.5 µg/ml) with or without Nec-1s (10 µM) for 24 h. After SYTOX Green staining, fluorescence intensity was quantified and normalized to TX-100-treated cells. **e** TNFα induces caspase-8 activation in RIPK1 S321A(A/A) mutant but not in WT and S321E(E/E) mutant MEFs. The MEFs were treated with TNFα (10 ng/ml) with or without Nec-1s (20 µM) for 24 h and caspase-8 activity was measured in the presence or absence of zVAD as described in Methods. Error bar, s.e.m. *, *t*-test *P* < 0.05; **P < 0.01, ***P < 0.001. Three independent repeats were included in each data point

To better understand the significance of RIPK1 S321 phosphorylation in regulating cell death, we generated knock-in MEFs carrying RIPK1 S321A or S321E mutations by CRISPR-Cas9 technology. Phosphorylation of S321 after TNFα stimulation was eliminated in S321A(A/A) MEFs (Supplementary Fig. 4a). S321A(A/A) or S321E(E/E) MEFs showed no difference in the phosphorylation of IKKα/β, or in the phosphorylation or degradation of IκBα in response to TNFα stimulation (Supplementary Fig. 4b), suggesting that S321 mutation has no effect on TNFα-stimulated NF-κB activation. Recruitment of CYLD, HOIP and Sharpin to TNF-RSC in response to TNFα was not affected in RIPK1 S321A(A/A) MEFs (Supplementary Fig. 4c). TNFα-induced RIPK1 recruitment and ubiquitination in TNF-RSC was slightly increased in RIPK1 S321A(A/A) MEFs (Supplementary Fig. 4d). On the other hand, homozygous S321A(A/A) MEFs showed increased sensitivity to cell death induced by TNFα, TNFα/cycloheximide (CHX) or TNFα/CHX/zVAD.fmk (zVAD) and Nec-1s protected S321A(A/A) MEFs to all three treatments (Fig. 5a–c). For better quantification of apoptosis, WT and S321A (A/A) MEFs were stained with SYTOX Green after TNFα alone or TNFα/CHX treatment. As shown in Fig. 5d and Supplementary Fig. 5a, S321A(A/A) MEFs were more sensitive than WT cells to apoptosis induced by TNFα or TNFα/CHX and Nec-1s only protected cell death in S321A(A/A) MEF cells. While TNFα/CHX normally promotes RIPK1-independent apoptosis in WT cells, cell death of RIPK1 S321A(A/A) MEFs induced by TNFα alone or TNFα/CHX was inhibited by Nec-1s, suggesting that the lack of S321 phosphorylation sensitizes cells to TNFα-induced RDA.

To verify the induction of RDA, we investigated the activation of caspases and RIPK1 in S321A(A/A) MEFs. We found that the activity of caspase-8 in S321A(A/A) MEFs, but not WT or S321E(E/E) MEFs, was stimulated by TNFα alone (Fig. 5e). Consistent with the activation of RDA, the addition of Nec-1s inhibited the activation of caspase-8. Following the evidence of enhanced caspase-8 activity, we examined the cleavage of RIPK1 and CYLD, two well-established substrates of caspase-8. Augmented cleavage of RIPK1 and CYLD was detected in S321A(A/A) compared to WT MEFs after TNFα/CHX treatment (Supplementary Fig. 5b). While the cleavage of CYLD and RIPK1 in both WT and S321A(A/A) MEFs was inhibited by pan-caspase inhibitor zVAD, the addition of Nec-1s was only able to inhibit the cleavage of CYLD and RIPK1 in S321A(A/A) MEFs, but not in WT MEFs, which further supports the induction of RDA in S321A(A/A) MEFs.

RIPK1 is known to be cleaved after D324 in human RIPK1 (its equivalent in murine RIPK1 is D325) by caspase-8 during TNFα mediated apoptosis^25^. Since S321 is only one amino acid beyond the usual 4-amino acid motif required for caspase recognition, we tested if the phosphorylation of S321 or its single point mutant might affect the cleavage by caspase-8 in vitro. However, S321A mutation had no effect on the cleavage by caspase-8 in vitro while S321E only showed weak increase of cleavage in vitro (Supplementary Fig. 5c). Thus, instead of determining the sensitivity of RIPK1 as a substrate for caspase-8, the phosphorylation of S321 is involved in the regulation of caspase-8 activation.

### RIPK1 S321A mutation promotes its binding with FADD

**Fig. 6:**
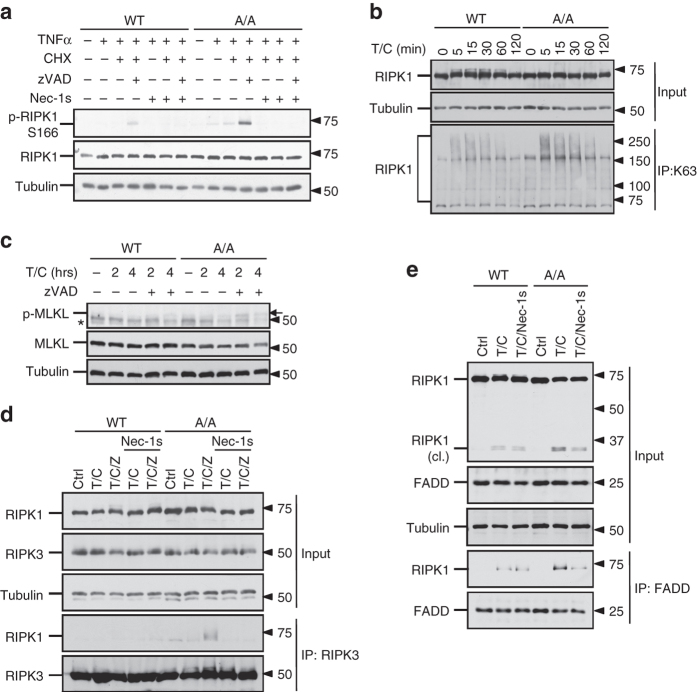
S321A mutation promotes RIPK1 activation. **a** RIPK1 S166 phosphorylation in S321A(A/A) mutant. WT and S321A(A/A) MEFs were treated with TNFα (50 ng/ml), CHX (1 µg/ml), zVAD (20 µM) and Nec-1s (10 µM) as indicated for 2 h. **b** S321A mutation augments RIPK1 K63 ubiquitination in response to TNFα/CHX treatment. MEFs were treated with TNFα (50 ng/ml) and CHX (1 µg/ml). Lysates under denaturing condition were collected at indicated time-points, immunoprecipitated with K63 antibody and detected with RIPK1 antibody. **c** Enhanced MLKL phosphorylation in S321A(A/A) cells in response to TNFα/CHX/zVAD. MEFs were treated with TNFα (10 ng/ml), CHX (1 µg/ml), zVAD (20 µM) for 2 and 4 h. Arrow, phosphorylated MLKL. **d** Earlier induction of RIPK1-RIPK3 interaction in S321A(A/A) MEFs induced by TNFα/CHX/zVAD. WT and RIPK1 S321A(A/A) MEFs were treated as **a** and co-immunoprecipitation was performed with RIPK3 antibody. **e** Stronger interaction between FADD and RIPK1 in RIPK1 S321A(A/A) MEFs induced by TNFα/CHX was suppressed by Nec-1s. The cells were treated with TNFα (50 ng/ml) and CHX (1 µg/ml) with or without Nec-1s (20 µM) for 4 h. Co-immunoprecipitation was performed with FADD antibody. *, nonspecific band

Phosphorylation of RIPK1 S166 has been established as a biomarker of RIPK1 activation^11, 26, 27^. To determine if the activation of RIPK1 might be affected by the phosphorylation of S321, we compared the phosphorylation of RIPK1 S166 in WT and S321A(A/A) MEFs stimulated by TNFα, TNFα/CHX or TNFα/CHX/zVAD. Stimulation of TNFα/CHX/zVAD is known to activate RIPK1 and necroptosis^26^. Consistently, the phosphorylation of RIPK1 S166 in WT MEFs was only detected upon stimulation by TNFα/CHX/zVAD together, but not by TNFα/CHX or TNFα alone (Fig. 6a). On the other hand, the phosphorylation of RIPK1 S166 in S321A(A/A) MEFs stimulated by TNFα/CHX/zVAD was significantly higher than that of WT, and furthermore, it was also detectable when stimulated by TNFα/CHX or TNFα alone (Fig. 6a). In addition, K63 ubiquitination of RIPK1 in TNFα-stimulated S321A(A/A) MEFs also increased (Fig. 6b). These results suggest that TNFα or TNFα/CHX treatment can promote the activation of RIPK1 when S321 phosphorylation is blocked. Given the increased caspase activity and RIPK1 S166 phosphorylation in S321A(A/A) MEFs, we concluded that blocking the phosphorylation of RIPK1 S321 sensitized cells to RDA upon stimulation by TNFα or TNFα/CHX, which was not sufficient to induce the activation of RIPK1 in WT cells.

Consistent with increased activation of RIPK1, S321A(A/A) MEFs also showed an increased sensitivity to necroptosis induced by TNFα/CHX/zVAD, which was inhibited by the addition of Nec-1s (Fig. 5a, c). On the other hand, the phosphorylation of MLKL and the interaction of RIPK1 and RIPK3 to form complex IIb, the hallmarks of necroptosis, were only detected in S321A(A/A) MEFs when stimulated by TNFα/CHX/zVAD, but not by TNFα/CHX (Fig. 6c, d). Thus, S321A mutation alone is not sufficient to activate necroptosis without caspase inhibition.

Since the activation of caspase-8 is mediated by the interaction of RIPK1 with FADD, we next characterized the interaction of RIPK1 and FADD in WT and S321A(A/A) MEFs by co-immunoprecipitation. We found that compared to that of WT MEFs, the interaction of RIPK1 and FADD in S321A(A/A) MEFs after treatment with TNFα/CHX was significantly increased. Furthermore, the treatment with Nec-1s inhibited the interaction of FADD and RIPK1 in S321A(A/A) MEFs but not in WT MEFs induced by TNFα/CHX (Fig. 6e). Taken together, we conclude that TAK1-mediated S321 phosphorylation on RIPK1 negatively regulates its activation and interaction with FADD. Thus, blocking S321 phosphorylation of RIPK1 sensitizes cells to TNFα-induced RDA by promoting the interaction of RIPK1 and FADD in a manner regulated by the kinase activity of RIPK1.

### RIPK1 hyperphosphorylation promotes necroptosis

**Fig. 7:**
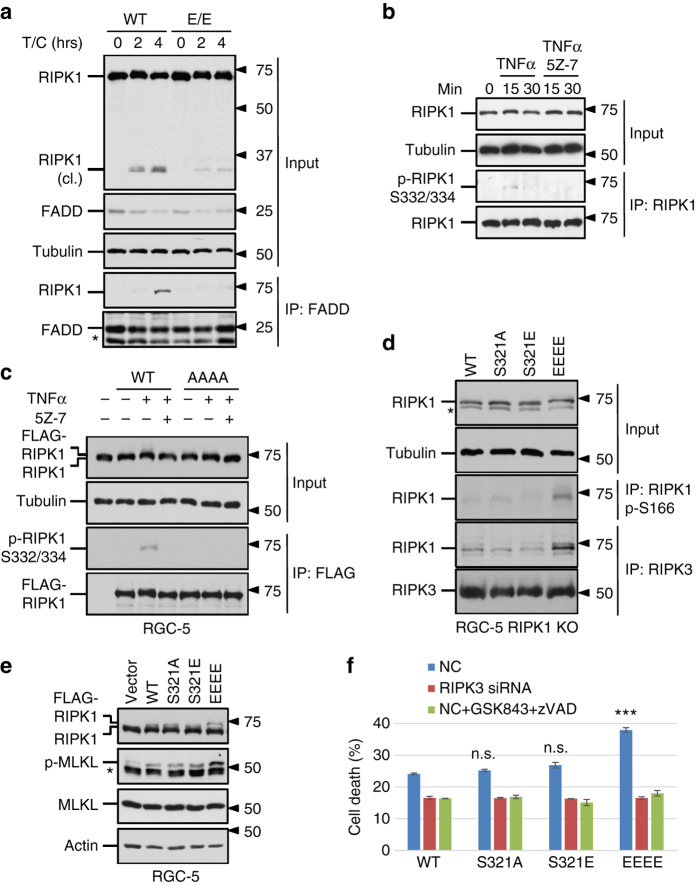
Hyperactivation of RIPK1 mediated by TAK1 sensitizes cells to necroptosis. **a** S321E mutation suppresses RIPK1 cleavage and RIPK1-FADD interaction induced by TNFα/CHX. The cells were treated with TNFα (50 ng/ml) and CHX (1 µg/ml) for 2 or 4 h. Co-immunoprecipitation was performed with FADD antibody. **b** TNFα induces RIPK1 S332/334 phosphorylation. RGC-5 cells were treated TNFα (10 ng/ml) with or without 5Z-7 (0.5 µM). RIPK1 was isolated by immunoprecipitation and detected by p-RIPK1 S332/334 and RIPK1 total antibodies. **c** RIPK1 S321/332/334/336A (AAAA) mutation blocks TNFα-induced S332/334 phosphorylation. FLAG-RIPK1 WT, AAAA mutant or empty vector was transiently expressed in RGC-5 cells and treated with TNFα (10 ng/ml) and 5Z-7 (0.5 µM) for 15 min. FLAG-RIPK1 was purified by anti-FLAG immunoprecipitation and detected by p-RIPK1 S332/334 and RIPK1 total antibodies. **d** RIPK1 kinase activity and its interaction with RIPK3 were induced by RIPK1 S321/332/334/336E (EEEE) mutant overexpression. RGC-5 RIPK1 KO cells were transfected with RIPK1 WT and mutants. 30 h after transfection, the cells were lysed and immunoprecipitated with RIPK1 p-S166 or RIPK3 antibodies and blotted with RIPK1 total antibody. **e** Transient expression of RIPK1 EEEE mutant promotes MLKL phosphorylation in RGC-5 cells. RGC-5 cells were transfected with FLAG-RIPK1 variants and samples were collected 24 h after transfection. **f** Spontaneous necroptosis in response to the expression of RIPK1 EEEE mutant. RGC-5 RIPK1 KO cells with or without RIPK3 knockdown were transfected with RIPK1 WT or mutants. GSK843 (10 µM)/zVAD (20 µM) was added as indicated. Cell death was measured by ToxiLight assay 24 h after transfection. *, nonspecific bands. Error bar, s.e.m. ***, *t*-test *P* < 0.001; n.s., not significant. Three independent repeats were included in each data point

Hyperactivation of TAK1 in TAB2 KO MEF has been shown to sensitize cells to necroptosis^8^; however, the mechanism is unclear. Given the enhanced phosphorylation on RIPK1 S321 in TAB2 KO cells, we hypothesized that hyperphosphorylation of RIPK1 by TAK1 might promote necroptosis. To test this hypothesis, we first characterized complex IIa formation and RIPK1 cleavage in S321E(E/E) MEFs induced by TNFα/CHX. When treated with TNFα/CHX, caspase-8-dependent RIPK1 cleavage was significantly reduced in S321E(E/E) MEF cells compared to WT (Fig. 7a). Furthermore, RIPK1 was co-immunoprecipitated with FADD in WT cells but not in S321E(E/E) mutant after 4 h TNFα/CHX treatment (Fig. 7a). Although the pro-apoptosis complex IIa was suppressed, RIPK1 S321E(E/E) MEFs were not more sensitive to TNFα/CHX/zVAD-induced necroptosis compared to WT cells (Fig. 5a). Thus, increased phosphorylation of RIPK1 S321 alone might not be sufficient to drive necroptosis.

RIPK1 can be phosphorylated in a number of Ser residues close to S321, e.g., S332 and S334 when expressed in 293T cells^11^. Since the phosphorylation of S332/334 was not affected by RIPK1 kinase-dead K45M mutation, they were unlikely to be sites of auto-phosphorylation. With additional S332/334/336A mutation, RIPK1 phosphorylation by recombinant TAK1 in in vitro kinase assay was further attenuated compared to kinase-dead RIPK1 S321A mutant (Supplementary Fig. 1e). To investigate the kinase mediating RIPK1 S332/334 phosphorylation, we generated an antibody against phosphorylation on these sites. Using this antibody, we found that the phosphorylation of S332/334 RIPK1 was also detectable in early time-points after TNFα stimulation and the signal was inhibited by 5Z-7, suggesting that they might also be phosphorylated by TAK1 (Fig. 7b). Furthermore, the signal detected by this p-S332/334 RIPK1 antibody was eliminated by S321/332/334/336A (AAAA) mutation (Fig. 7c).

**Fig. 8:**
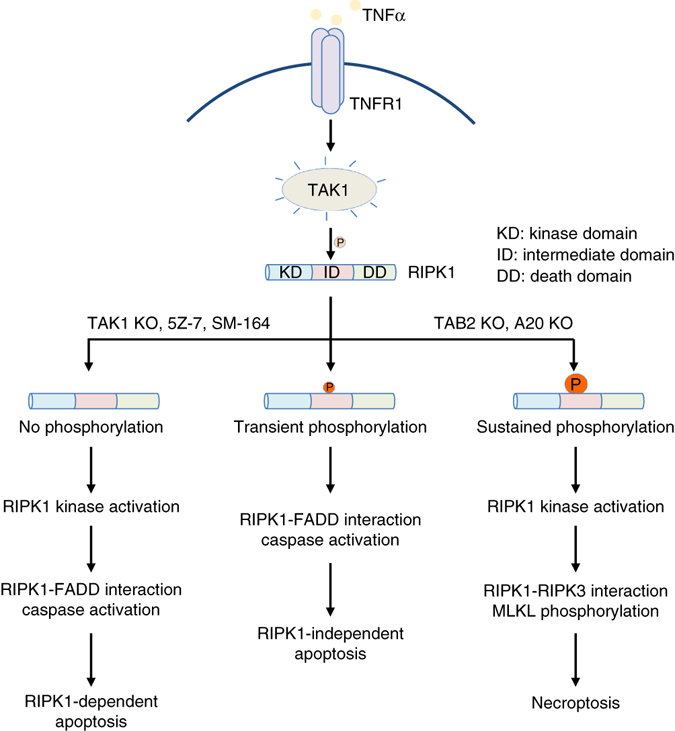
TAK1-mediated RIPK1 phosphorylation dictates the activation multiple cell death pathways. Upon TNFα treatment, TAK1 is activated to mediate RIPK1 phosphorylation on the intermediate domain. Under normal conditions, TNFα/CHX induces transient phosphorylation of RIPK1 S321 by TAK1 and leads to RIPK1-independent apoptosis. Dysregulation of RIPK1 phosphorylation by TAK1 promotes its activation. When the phosphorylation of RIPK1 intermediate domain is blocked as in TAK1 KO cells, with inhibitor of TAK1 or IAP antagonist (SM-164), TNFα alone or TNFα/CHX treatment promotes the activation of RIPK1 and its binding with FADD to mediate RDA. On the other hand, hyperphosphorylation of RIPK1 intermediate domain, as in TNFα-stimulated TAB2 KO and A20 KO cells, promotes its interaction with RIPK3 to mediate necroptosis

To characterize the effect of additional TAK1 phosphorylation sites on RIPK1, we mutated these three TAK1 sites together with S321 to generate RIPK1 S321/332/334/336E (EEEE) quadruple mutant as a model for sustained RIPK1 phosphorylation in the intermediate domain. We found that the expression of RIPK1 EEEE mutant in RGC-5 RIPK1 KO cells was sufficient to induce spontaneous RIPK1 kinase activation as shown by the detection of RIPK1 S166 phosphorylation and its interaction with RIPK3 in the absence of TNFα stimulation (Fig. 7d). Increased phosphorylation of MLKL was detected in RGC-5 cells expressing RIPK1 EEEE mutant (Fig. 7e). As a result, RIPK1 EEEE mutant induces spontaneous cell death which could be protected by RIPK3 knockdown or treatment of RIPK3 inhibitor (Fig. 7f). The expression level of RIPK1 EEEE mutant was comparable to that of other RIPK1 variants (Fig. 7d, e and Supplementary Fig. 6b), so the activation of RIPK1 and RIPK3 as well as consequent cells death were not due to higher expression of RIPK1 EEEE mutant. Taken together, these results suggest that sustained RIPK1 phosphorylation in the intermediate domain promotes its interaction with RIPK3 to drive necroptosis (Fig. 8).

## Discussion

In this manuscript, we demonstrate a novel mechanism by which phosphorylation of the intermediate domain of RIPK1 by TAK1 dictates alternative cell death mechanisms. We show that dysregulation of RIPK1 phosphorylation by TAK1, including both inhibition or hyperphosphorylation, promotes the activation of RIPK1. On the other hand, TAK1-mediated phosphorylation in the intermediate domain of RIPK1 dictates whether RIPK1 interacts with FADD to form the complex IIa to mediate apoptosis in RIPK1-dependent or -independent manner, or with RIPK3 to form the necrosome (complex IIb) to drive necroptosis. Specifically, the lack of RIPK1 S321 phosphorylation by TAK1 promotes its interaction with FADD to mediate RDA; whereas excessive phosphorylation of RIPK1 by TAK1 in the intermediate domain promotes its interaction with RIPK3 while suppressing its binding with FADD to promote necroptosis. In WT cells, TNFα-induced transient phosphorylation of RIPK1 by TAK1 in the presence of CHX is sufficient to block the activation of RIPK1 and drives RIPK1-independent apoptosis. On the other hand, the absence of S321 phosphorylation on RIPK1 in TNFα alone or TNFα/CHX-stimulated S321A(A/A) cells promotes the activation of RIPK1 and RIPK1-dependent caspase activation to mediate RDA. Finally, the sustained phosphorylation of RIPK1 by TAK1 modeled by RIPK1 EEEE mutant suppresses RIPK1-FADD interaction but promotes the interaction of RIPK1 and RIPK3 to promote the activation of necroptosis in the absence of TNFα stimulation. Thus, absent, transient and sustained levels of TAK1-mediated RIPK1 phosphorylation in TNF-RSC may represent three distinct states to dictate the execution of three alternative cell death mechanisms, RDA, RIPK1-independent cell death and necroptosis, by regulating the interaction of RIPK1 with FADD in kinase-dependent or -independent manner to mediate apoptosis, or with RIPK3 to mediate necroptosis (Fig. 8).

In TNFα-stimulated cells, TAK1 is recruited to TNFR1 in a RIPK1-dependent manner to promote the phosphorylation of I*κ*B kinase (IKK) and activation of NF-κB pathway, which has a powerful pro-survival role by inducing the expression of target genes that can block apoptosis, promote cell proliferation and stimulate inflammatory responses^6, 28, 29^. Our study defines a novel molecular contribution of TAK1 that controls the activity of RIPK1 kinase in mediating apoptosis, which is consistent, but distinct, from the role of TAK1 in promoting cell survival and inflammation in mediating NF-κB pathway activation. Supporting this idea, S321A mutation has no effect on the activation of NF-κB when cells are stimulated by TNFα, as blocking NF-κB activation by the addition of CHX, Smac mimetic, or TAK1 inhibitor, 5Z-7, is still required to significantly induce apoptosis in S321A(A/A) MEFs.

Our results demonstrate that deficiencies in TAB2 or A20 promote the phosphorylation of RIPK1 S321. Elevated levels of RIPK1 phosphorylation in the intermediate domain in TAB2 KO MEFs drive the interaction of RIPK1 and RIPK3 to promote necroptosis. On the other hand, A20, encoded by the gene *TNFAIP3*, is an important ubiquitin-editing enzyme recruited to TNF-RSC to terminate multiple downstream events including NF-κB-mediated transcriptional response, RIPK1-mediated signaling and ultimately, the disassembly of the TNFR1 signaling complex^21, 30^. K63 ubiquitination of RIPK1 is abnormally elevated in A20-deficient cells^31^. Since K63 ubiquitination of TNF-RSC is critical for mediating the activation of TAK1^4^, the defective removal of K63 ubiquitination from TNF-RSC in A20-deficient cells are predicted to lead to sustained TAK1 activation. However, since increased activation of TAK1 in A20-deficient cells is expected to promote sustained activation of NF-κB, it has been puzzling as to why A20 deficiency also sensitizes cells to necroptosis^7^. Our study reveals a previously unexpected link between A20-regulated ubiquitination of TNF-RSC and TAK1-mediated RIPK1 phosphorylation, which provides mechanistic insights as how A20 deficiency might promote the activation of RIPK1/RIPK3 complex to mediate necroptosis independent of NF-κB activation. Recent genome-wide association studies have identified single-nucleotide polymorphisms at the TNFAIP3/A20 locus in humans that are linked to susceptibility/resistance to inflammatory and autoimmune diseases^32^. Our study also suggests the possible clinical application of phospho-S320 in human RIPK1 (equivalent to S321 in murine RIPK1) as a biomarker for RIPK1-dependent cell death and inflammation.

## Methods

### Reagents antibodies and cell lines

The following commercial antibodies and reagents were used in this study: TAK1, Cell Signaling Technology (5206); RIPK1, Cell Signaling Technology (3493) and BD Biosciences (610459); TBK1, Cell Signaling Technology (3504); p-IKKα/β, Cell Signaling Technology (2697); IKKβ, Cell Signaling Technology (8943); IκBα, Santa Cruz (sc-371); p-p38, Cell Signaling Technology (9211); p38, Cell Signaling Technology (9212); CYLD, Cell Signaling Technology (8462); FADD, Abcam (ab124812) and Santa Cruz (6036); α-Tubulin, Sigma-Aldrich (T9026); β-actin, Santa Cruz (81178). α-Tubulin and β-actin antibodies were used with 5000-fold dilution and other antibodies were used with 1000-fold dilution. Uncropped scans of the most important blots were provided as Supplementary Fig. 7 in the Supplementary Information. 5Z-7 was from Sigma-Aldrich (O9890). Recombinant TAK1-TAB1 and GST-IKKα fusion proteins were obtained from Millipore (14-600) and Sigma (SRP5040). CellTiter-Glo luminescent cell viability kit was from Promega. 7-Cl-O-Nec-1 (Nec-1s) was made by custom synthesis. L929 and BV-2 cells were purchased from ATCC. TRAF2 and TRADD KO MEFs were provided by Dr. Zhenggang Liu. cIAP1/2 DKO MEFs were provided by Dr. John Silke. TAB2 KO MEFs were provided by Dr. Jun Ninomiya-Tsuji. Cell lines used in this study were tested every 3 months for mycoplasma contamination by MycoAlert Mycoplasma Detection Kit from Lonza.

### Generation of anti-p-RIPK1 S321 and S332/334 antibodies

The phospho-peptides, VLQRMFpSLQHDC (S321) and CVPLPPpSRpSNSEQPG (S332/334), were synthesized and coupled to KLH carrier protein via Cys at the C terminus. Polyclonal anti-p-S321 or p-S332/334 RIPK1 antibodies were produced in rabbits against p-peptide antigen by ProteinTech.

### AAV-RIPK1 vector and virus production

rAAV vector plasmids carrying the vector genomes with WT, S321A or S321E RIPK1 gene expression cassettes under the control of human thyroxine binding globulin (TBG) promoter, which directs efficient and sustaining transgene expression in liver-specific pattern^24^, are transfected individually into HEK293 cells with a packaging plasmid and adenovirus helper plasmid. The recombinant viruses were purified by standard CsCl gradient sedimentation method and desalted by dialysis^33^. The quality of vectors was tested by qPCR titration for DNase resistant vector genome concentration, silver-stained SDS-polyacrylamide gel analysis to establish the purity of each lot, electron microscopic analysis to independently test full/empty virus ratios^33^.

### Generation of TAK1^F/F^ and RIPK1 mutant MEFs

Super-ovulated female B6D2F1 mice (7–8 weeks old) were mated to B6D2F1 vasectomy males, and zygotes were collected from oviducts. Cas9 mRNA and sgRNA were prepared by in vitro transcription. Single-stranded oligonucleotides (ssODN) were synthesized by Sangon. 100 ng/μl Cas9 mRNA, 50 ng/μl sgRNA and 100 ng/μl ssODN were mixed in M2 medium (Sigma) and injected into the cytoplasm of zygote using a microinjector (FemtoJet, Eppendorf). The injected zygotes were cultured in KSOM with amino acids at 37 °C under 5% CO_2_ in air until transplantation. Thereafter, 20–25 injected embryos were transferred into oviducts of pseudopregnant ICR females at 0.5 d.p.c. The pups were identified by both enzyme digestion and DNA sequencing. The positive mice were mated with WT C57BL/6 mice to produce heterozygous S321A or E mutant progenies, which are then backcrossed with C57BL/6 mice.

Target sequence:

5′-TTTGACCTGCTCGGAGGTAA-3′

S321A ssODN (the synonymous mutation were highlighted by bold and point mutations were highlighted by Italic):

5′-cattacagaaagagtatccagatcaaagcccagtgctgcagagaatgttt*GCA* **TTG**CAGCATGACTGTGTACCC**TTGCCG**ccgagcaggtcaaattcaggtaactcacctattcgttcatttgcatactc-3′

S321E ssODN:

5′-cattacagaaagagtatccagatcaaagcccagtgctgcagagaatgttt*GAA* **TTG**CAGCATGACTGTGTACCC**TTGCCG**ccgagcaggtcaaattcaggtaactcacctattcgttcatttgcatactc-3′

The founder mice carrying S321A or S321E mutant were backcrossed with C57BL/6 mice to produce heterozygous mice. WT and homozygous S321A(A/A) or WT and homozygous S321E(E/E) embryonic fibroblast cells were isolated from littermates of E13.5 embryos of heterozygous crosses. All experiments on mice were conducted according to the protocols approved by the Harvard Medical School Animal Care Committee and Institutional Animal Care and Use Committee of the Interdisciplinary Research Center on Biology and Chemistry of Chinese Academy of Sciences.

TAK1^F/F^ MEFs were immortalized spontaneously in culture and infected by virus for the expression of Cre recombinase to generate TAK1-deficient MEF. RIPK1 S321A and S321E MEFs were immortalized by viral infection and subsequent expression of SV-40 large T antigen.

### Caspase activity assay

Cell lysates were collected and mixed with substrate for ﻿caspase-8 (caspase-Glo 8 Assay, Promega) and the activity was determined according to the manufacturer’s instructions.

### In vitro caspase-8 cleavage assay

RIPK1 WT, S321A and S321E plasmids were transcribed and translated in vitro with [^35^S]-methionine labeling using TNT T7 Quick coupled transcription/translation kit (Promega). In vitro cleavage assay was performed in cleavage buffer containing 20 mM HEPES (pH = 7.4), 100 mM NaCl, 20 mM dithiothreitol and 0.5% NP-40 at 37 °C for 1 h^34^.

### In vitro kinase assay

Kinase-dead FLAG-RIPK1 variants with or without additional mutations or kinase-dead IKKβ were transiently expressed in 293T and immunoprecipitated with anti-FLAG M2-agarose beads. Protein bound FLAG beads were incubated with 100 ng recombinant TAK1 in kinase buffer containing 20 mM HEPES (pH = 7.3), 10 mM MgCl_2_, 10 mM MnCl_2_, 10 µM cold ATP and 1 µCi of [γ-^32^P]ATP^11^. When indicated in the text, 1 µM 5Z-7 was added to the reaction mixture. The reactions were carried out at 30 °C for 60 min and stopped by boiling in SDS-PAGE loading buffer at 95 °C for 5 min. Similar protocol was used for RIPK1 S321A phosphorylation assay. To confirm anti-p-S321-RIPK1 antibody specificity, 293T cells transiently expressing FLAG-RIPK1 WT or S321A were treated with 5Z-7 (0.5 µM) and Nec-1s (10 µM) to minimize RIPK1 phosphorylation background. RIPK1 WT and S321A mutant were purified and incubate with recombinant TAK1 or IKKα in kinase buffer without [γ-^32^P]ATP at 30 °C for 30 min.

### Statistics

Data are expressed as the mean ± s.e.m. Error bar, s.e.m. Pairwise comparisons between two groups were performed using the Student’s *t*-test. Differences were considered statistically significant if *P* < 0.05 (*), *P* < 0.01 (**), *P* < 0.001 (***) or not significant (n.s.). At least three independent biological repeats were included in each data point. Each experiment was repeated at least three times.

### Data availability

The authors declare that the data supporting the findings of this study are available within the paper and its Supplementary Information Files.

## Electronic supplementary material

Supplementary Information
