# Supplementary material for: Regulation of RIPK1 activation by TAK1-mediated phosphorylation dictates apoptosis and necroptosis
Source: Nat Commun. 2017 Aug 25;8:359. doi: 10.1038/s41467-017-00406-w (PMC5572456; doi:10.1038/s41467-017-00406-w)
Supplement: Supplementary file 1 — Supplementary Information [file 41467_2017_406_MOESM1_ESM.pdf]

Description of Supplementary Files

Title: Supplementary Information

Description: Supplementary Figures

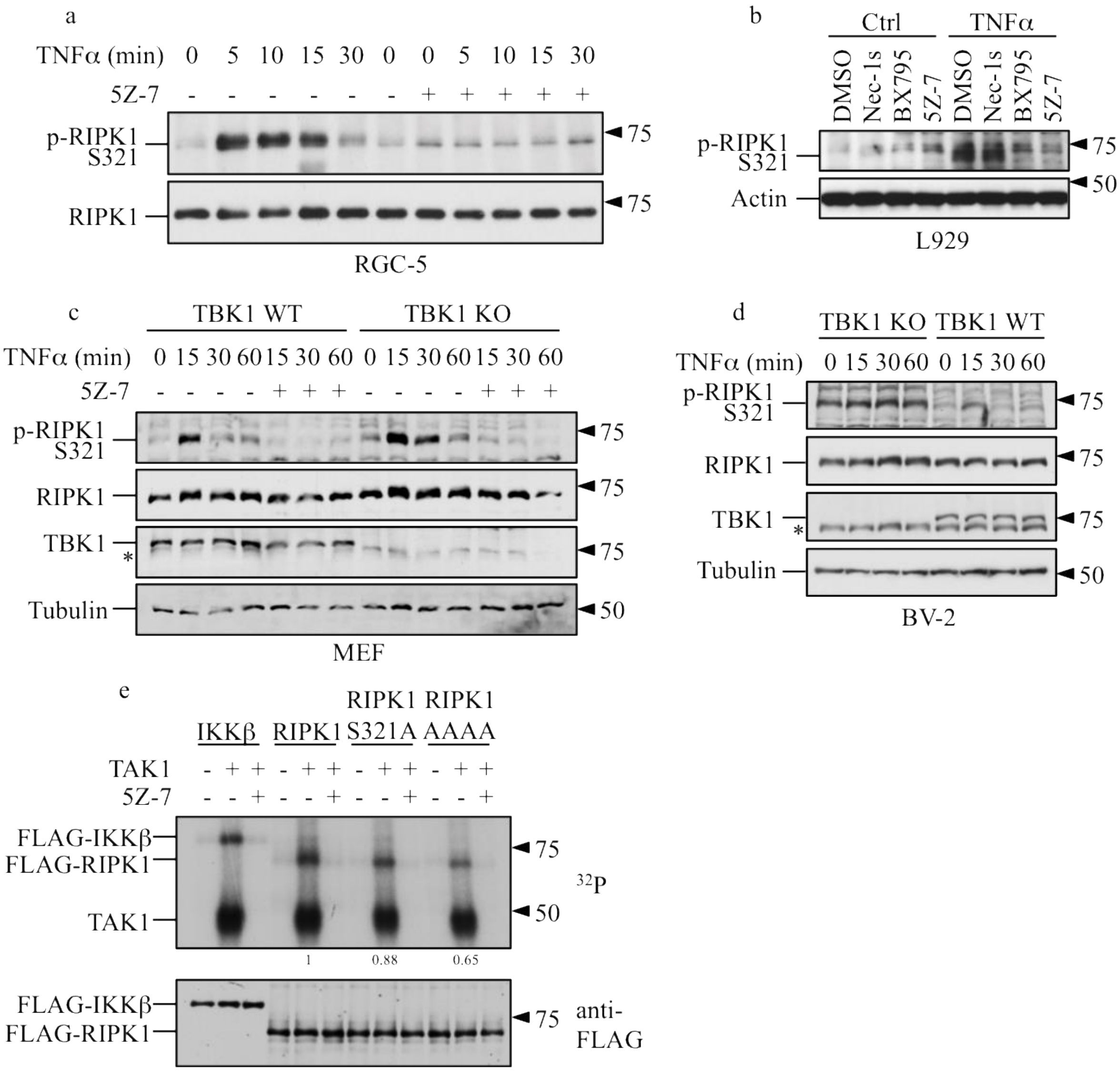

Supplementary Figure 1. TAK1 but not TBK1 mediates TNFα-induced RIPK1 S321 phosphorylation. (a) TNFα induces RIPK1 S321 phosphorylation in RGC-5 cells. RGC-5 cells treated with TNFα (20 ng/ml) with or without 5Z-7 (0.5 µM). The samples were immunoprecipitated with RIPK1 antibody and analyzed by anti-p-S321-RIPK1 and total RIPK1 antibodies. (b) Effect of TAK1 and TBK1 inhibitors on RIPK1 S321 phosphorylation in L929 cells. L929 cells were treated by TNFα (20 ng/ml) together with DMSO, Nec-1s (10 µM), TBK1 inhibitor, BX795 (1 µM) and 5Z-7 (0.5 µM). (c-d) TBK1 KO in MEFs or BV-2 cells did not block RIPK1 S321 phosphorylation induced by TNFα. The cells were treated with TNFα (10 ng/ml) with or without 5Z-7 (0.5 µM). (e) TAK1 phosphorylates multiple sites on RIPK1 *in vitro*. Kinase-dead FLAG-IKKβ and FLAG-RIPK1 with indicated point mutations were transiently expressed in 293T cells and purified with anti-FLAG immunoprecipitation as substrates in kinase assay. Substrates, recombinant TAK1-TAB1 fusion protein and 5Z-7 were mixed as indicated. Aliquot of mixtures was blotted with FLAG antibody to confirm IKKβ and RIPK1 loading amount and the radioactive kinase assay in the presence of ^{32}P-ATP was performed as described in Methods. The numbers at the bottom of top panel were relative ratio of phosphorylated RIPK1 to autophosphorylated TAK1. *, non-specific bands.

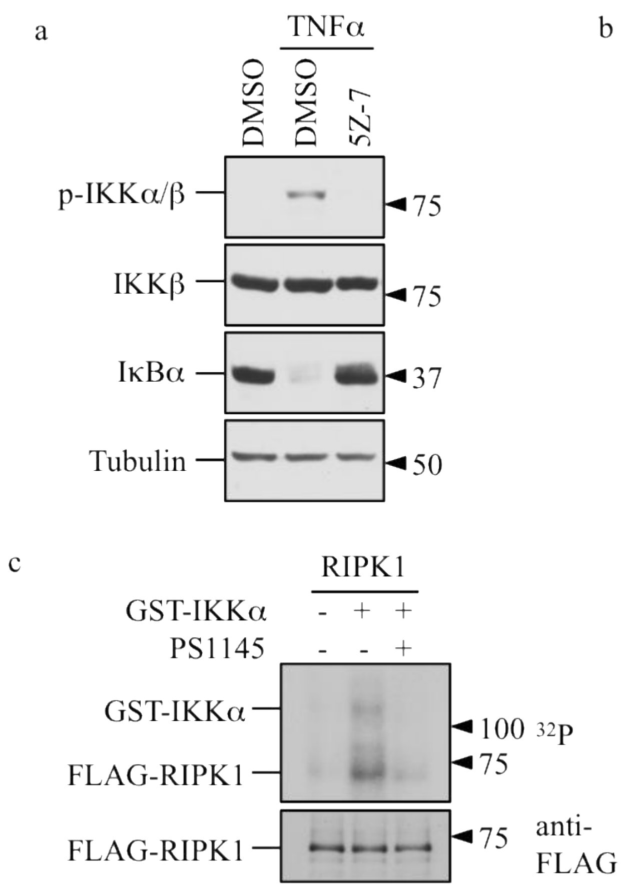

Supplementary Figure 2. Phosphorylation of RIPK1 S321 induced by TNFα is not blocked by IKKα/β inhibition. (a) TAK1 inhibitor, 5Z-7, blocks IKKα/β phosphorylation induced by TNFα. L929 cells were treated with TNFα (10 ng/ml) for 5 min in the presence or absence of 5Z-7 (0.5 μM). (b) IKKα/β inhibitor, BMS345541, does not block RIPK1 S321 phosphorylation. MEFs were treated with TNFα (10 ng/ml) with or without BMS345541 (10 μM) and the samples were collected at indicated time points. (c) IKKα phosphorylates RIPK1 *in vitro*. *In vitro* radioactive kinase assay were performed in the presence of ^{32}P-ATP with purified kinase dead FLAG-RIPK1 and recombinant GST-IKKα. Aliquot of kinase reaction mixtures was blotted with FLAG antibody to confirm the RIPK1 loading amount.

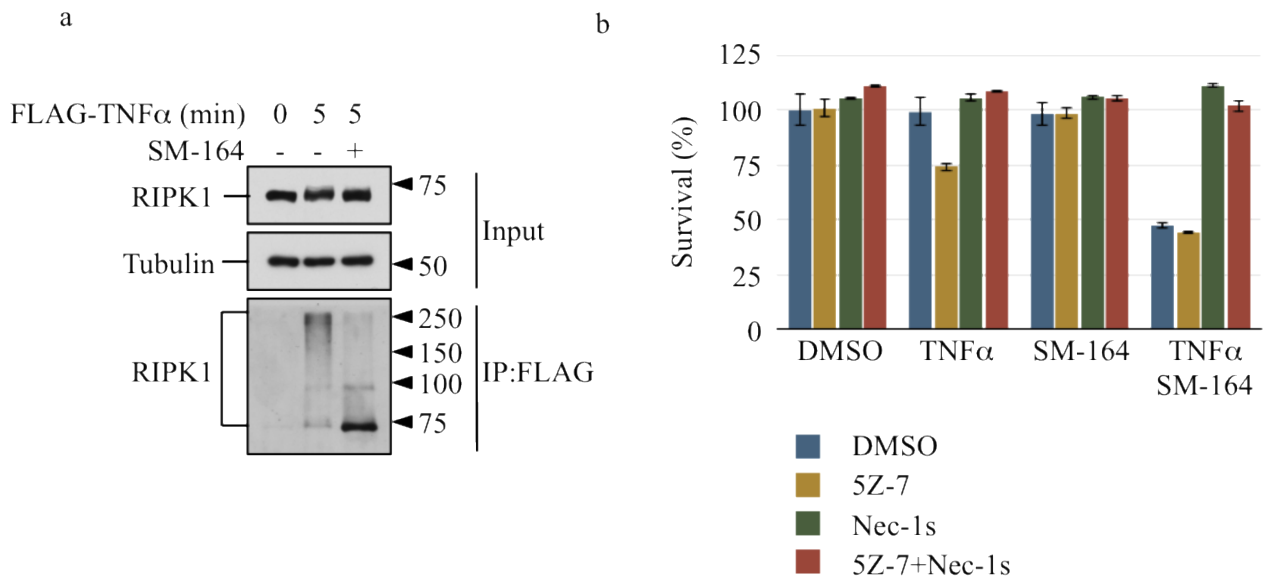

Supplementary Figure 3. Sensitization of RDA by SM-164. (a) SM-164 treatment inhibits TNFα-induced RIPK1 ubiquitination. MEFs were pre-incubated with DMSO or SM-164 (50 nM) for 4 h and treated with FLAG-TNFα (50 ng/ml) for 5 min. TNF-RSC was purified by anti-FLAG co-immunoprecipitation and detected with RIPK1 antibody. (b) TAK1 inhibitor does not further sensitize SM-164 pretreated cells to TNFα-induced cell death. The cells were pre-treated for SM-164 (50 nM) for 4 h, then with 5Z-7 (0.5 µM) and/or Nec-1s (10 µM) for 30 min. Then the cells were incubated with TNFα for 1.5 h and the viability was determined by CellTiter-Gro. Error bar, SEM.

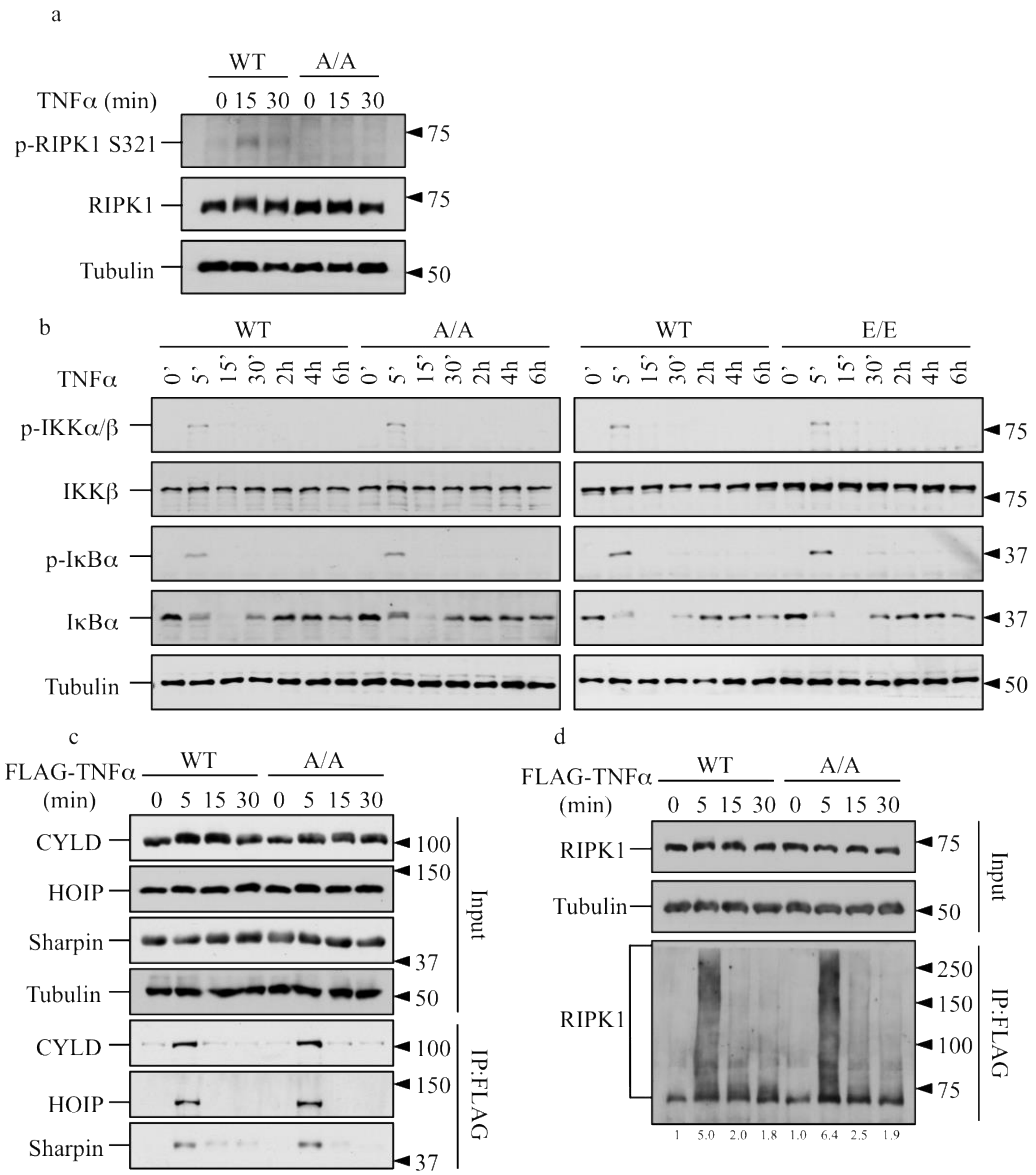

Supplementary Figure 4. Effect of RIPK1 S321A mutation on NF-κB activation and TNF-RSC formation. (a) Blockage of RIPK1 S321 phosphorylation in RIPK1 S321A (A/A) MEFs. WT and A/A MEFs were treated with TNFα (10 ng/ml) and the samples collected at indicated time-points were analyzed by western blot using indicated antibodies. (b) RIPK1 S321A or S321E mutation does not affect NF-κB pathways. WT, RIPK1 S321A(A/A) and S321E(E/E) MEFs were stimulated by TNFα (10 ng/ml) for indicated period of time. The phosphorylation of IKKα/β and IκBα and degradation of IκBα were determined by western blot using indicated antibodies. (c-d) TNF-RSC in RIPK1 S321A(A/A) MEFs. MEFs were treated with FLAG-TNFα (50 ng/ml) for 5, 15 and 30 min. TNF-RSC was isolated by anti-FLAG co-immunoprecipitation and detected with indicated antibodies.

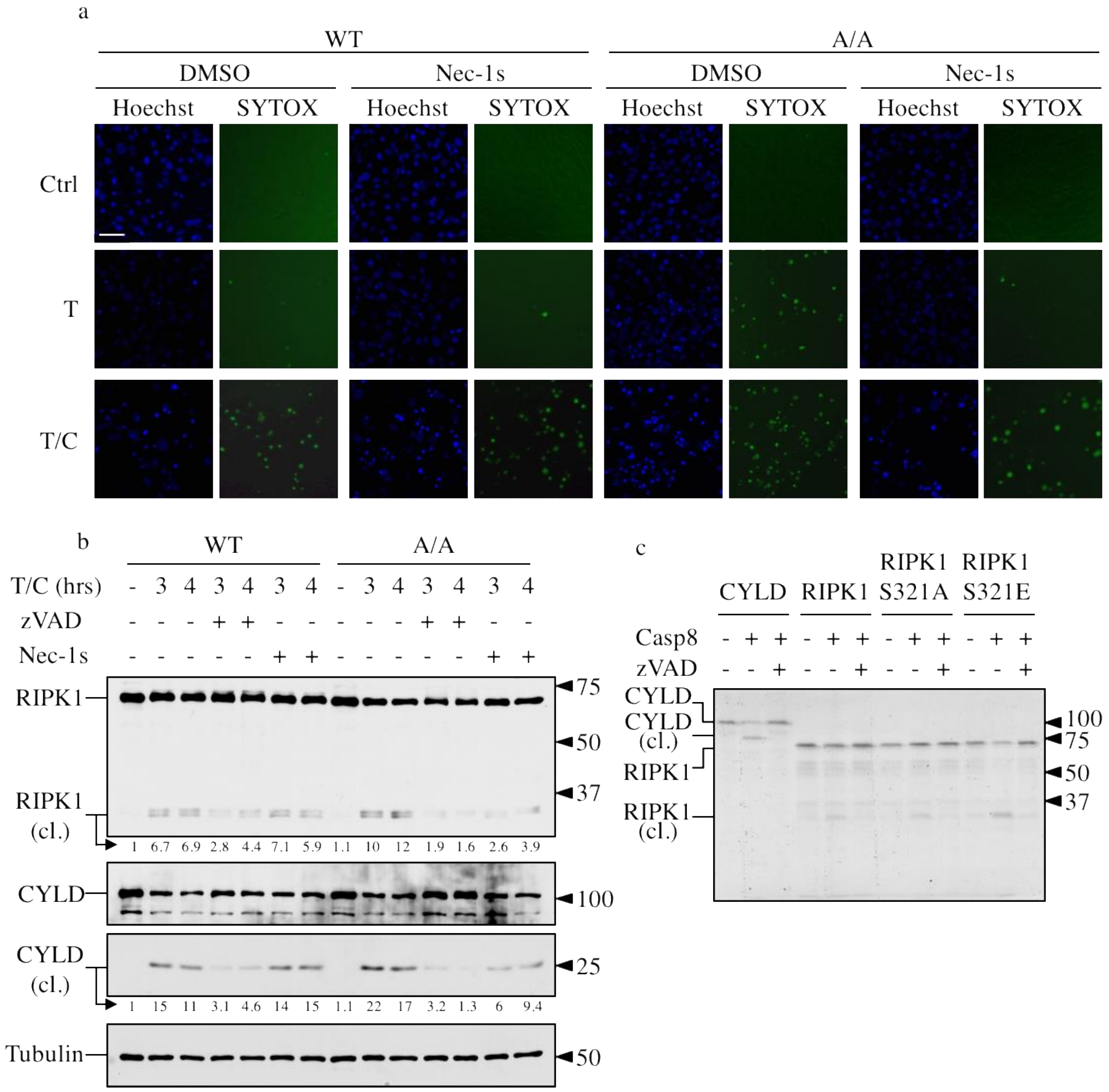

Supplementary Figure 5. Sensitization of RDA by RIPK1 S321A mutation. (a) TNFα alone or TNFα/CHX induces RDA in RIPK1 S321A(A/A) mutant MEFs. WT and RIPK1 S321A(A/A) MEFs were treated with TNFα (10 ng/ml) or CHX (0.5 µg/ml) with or without Nec-1s (10 µM) for 24 h. After SYTOX Green and Hoechst 33342 staining, images were collected by fluorescence microscopy. Bar, 50 µm. (b) Nec-1s inhibits the enhanced cleavage of RIPK1 and CYLD in RIPK1 S321A(A/A) MEFs after TNFα/CHX treatment. WT and RIPK1 S321A(A/A) MEFs were treated with TNFα (50 ng/ml) and CHX (1 µg/ml) with or without Nec-1s (20 µM) or zVAD (20 µM). Relative intensity of cleaved RIPK1 and CYLD bands was quantified. (c) RIPK1 S321A/E point mutations do not affect the cleavage by caspase-8 *in vitro*. RIPK1 WT, S321A, and S321E proteins were synthesized *in vitro* using ^{35}S-Met labeling and incubated with recombinant active caspase-8 with or without zVAD at 37 ˚C for 1 h. CYLD, a known caspase-8 substrate, was used as a positive control.

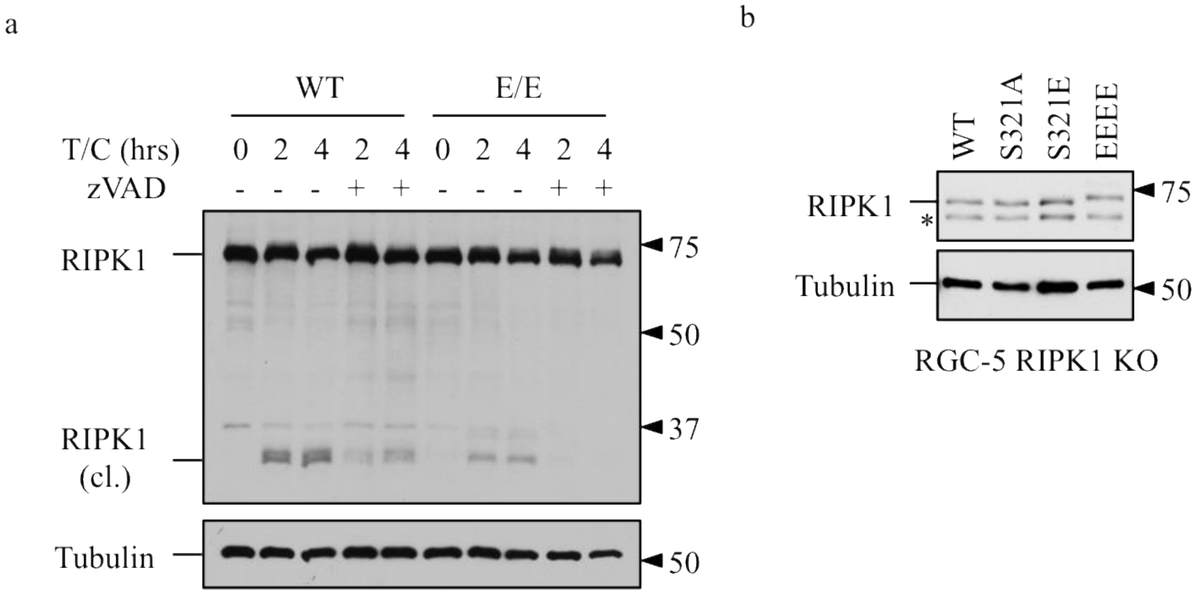

Supplementary Figure 6. Reduced RIPK1 cleavage in RIPK1 S321E(E/E) MEF. (a) WT and RIPK1 S321E(E/E) MEFs were treated with TNFα (50 ng/ml) and CHX (1 µg/ml) with or without zVAD (20 µM). The samples were collected at indicated time-points. (b) Expression level of RIPK1 WT and mutants. FLAG-RIPK1 WT, S321A, S321E or S321/332/334/336E (EEEE) was transiently expressed in RGC-5 RIPK1 KO cells. Samples were collected 24 h after transfection. *, non-specific band.

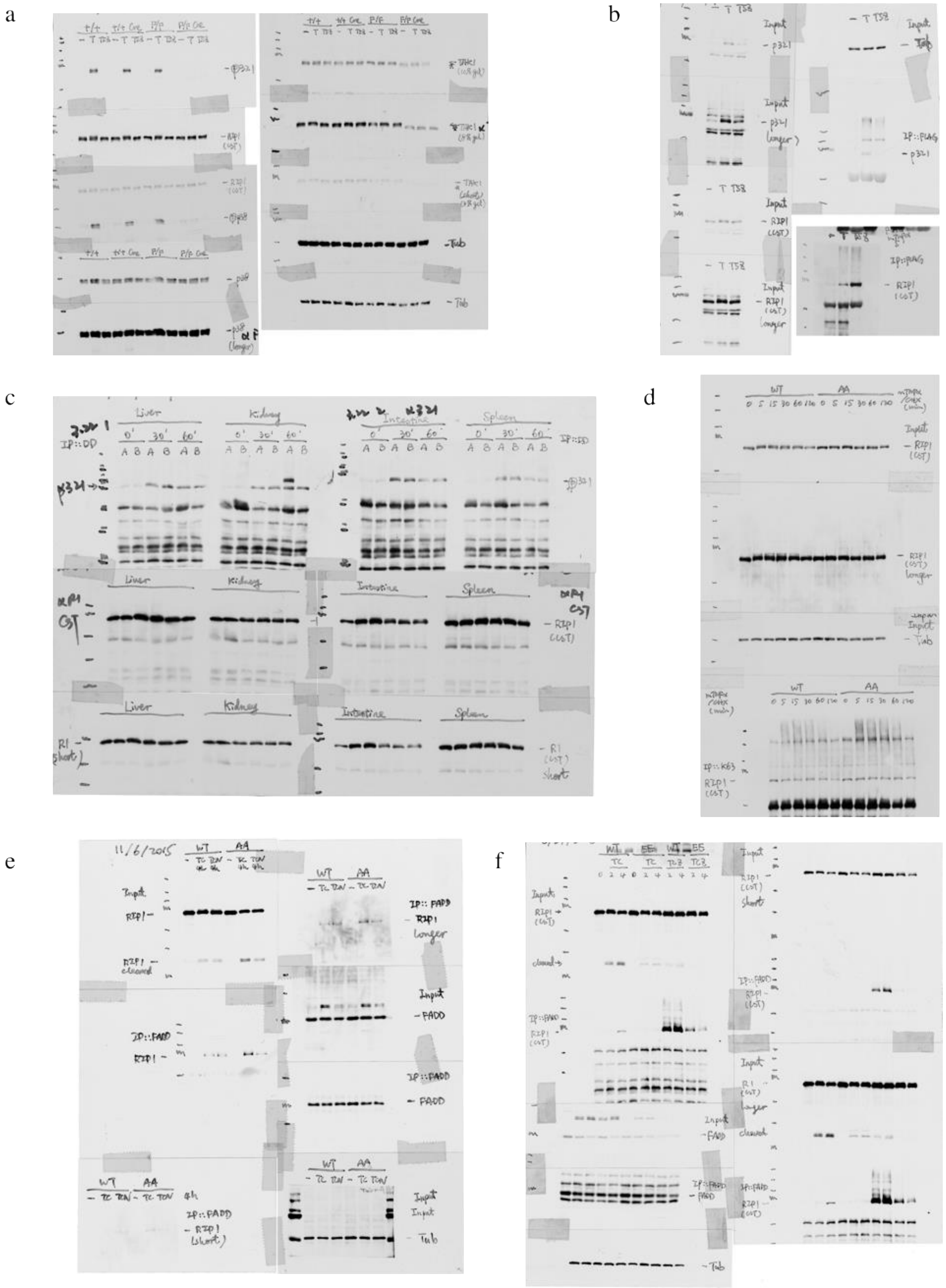

Supplementary Figure 7. Uncropped scans of important blots. (a) Fig. 2b. (b) Fig. 2c. (c) Fig. 4a. (d) Fig. 6b. (e) Fig. 6e. (f) Fig. 7a.
